# Coordination of miR-192 and miR-22 in p53-Mediated Cell Fate Decision

**DOI:** 10.3390/ijms20194768

**Published:** 2019-09-26

**Authors:** Cheng-Yuan Sun, Xiao-Peng Zhang, Wei Wang

**Affiliations:** 1 National Laboratory of Solid State Microstructure and Department of Physics, Nanjing University, Nanjing 210093, China; 13770503372@163.com; 2 Kuang Yaming Honors School, Nanjing University, Nanjing 210023, China; 3 Institute for Brain Sciences, Nanjing University, Nanjing 210023, China

**Keywords:** p53-inducible microRNA, cell cycle arrest, apoptosis

## Abstract

p53-targeted microRNAs (miRNAs) markedly affect cellular response to DNA damage. These miRNAs may contribute to either cell cycle arrest or apoptosis induction. However, how these miRNAs coordinate to modulate the decision between cell survival and death remains less understood. Here, we developed an integrated model of p53 signaling network to investigate how p53-targeted *miR-192* and *miR-22* modulate cellular outcome in response to DNA damage. By numerical simulations, we found that p53 is activated progressively depending on the extent of DNA damage. Upon moderate damage, p53 rises to medium levels and induces *miR-192* to promote its own activation, facilitating *p21* induction and cell cycle arrest. Upon severe damage, p53 reaches high levels and is fully activated due to phosphatase and tensin homolog (PTEN) induction. As a result, it transactivates *miR-22* to repress *p21* expression and activate E2F1, resulting in apoptosis. Therefore, miR-192 promotes primary activation of p53, while miR-22 promotes apoptosis by downregulating p21. This work may advance the understanding of the mechanism for cell fate decision between life and death by p53-inducible miRNAs.

## 1. Introduction

p53 is a famous tumor suppressor that plays a key role in maintaining genomic integrity and preventing malignant transformation [1]. It can be activated by various cellular stresses including DNA damage, oncogene activation, hypoxia and replicative stress [2]. It mainly acts as a potent transcription factor to induce a large number of target genes involved in cell cycle arrest, senescence or apoptosis [3,4]. The underlying mechanism for p53-mediated cell fate decision is not fully understood.

Selective expression of p53-targeted genes is crucial for the decision between survival and death in response to DNA damage [5,6]. Among the products of these genes, p21 can induce cell cycle arrest [7], while Bax contributes to p53-dependent apoptosis [8]. p21 can also protect damaged cells from apoptosis [9]. It is downregulated through several mechanisms during apoptosis induction. On one hand, degradation of p53 cofactor Hzf represses *p21* expression in response to prolonged or severe damage [10,11]. On the other hand, p53 induces *damaged-DNA binding protein 2* (*DDB2*) to promote p21 degradation upon lethal damage [12,13]. It is intriguing to reveal how *p21* expression is regulated dynamically to induce cell cycle arrest or apoptosis in the DNA damage response (DDR).

p53 dynamics also modulate cell fate decision in the DDR. In cells exposed to γ-radiation, when p53 dynamics are changed from pulsing to sustained activation, cell fate shifts from cell cycle arrest to senescence [14]. Our modeling work showed that p53 exhibits two-phase dynamics in response to ionizing radiation: it undergoes a number of pulses followed by sustained high levels, and induces proarrest and proapoptotic genes in the early and late phase, respectively [15]. Notably, the two-phase dynamics of p53 have been verified in MCF-7 cells treated with genotoxic drugs [16]. However, p53 cannot show pulses in response to UV radiation or other DNA damaging agents such as cisplatin [17]. In the absence of pulses, how p53 dynamics modulate the decision between life and death is less understood.

Notably, p53 also mediates the production of microRNAs (miRNAs) to repress the expression of some genes post-transcriptionally in cellular response to DNA damage [18]. First, p53 induces the expression of several miRNAs including *miR-22*, *miR-34*, *miR-145*, *miR-192* and *miR-605* to modulate the cellular response [19,20,21,22]. Second, it promotes the processing and maturation of some miRNAs including miR-16, miR-143 and miR-145 [23]. Third, it represses some other miRNAs like *miR-17-92* to promote apoptosis in response to hypoxia [24,25]. p53-mediated miRNAs have become important components of the p53 network and increase the complexity of the network [26]. It is necessary to explore how p53-targeted miRNAs influence p53 dynamics and cellular outcome.

p53-mediated miRNAs play different roles in the DDR. On one hand, miR-143/145, miR-192 and miR-605 modulate p53 dynamics by repressing Mdm2 translation [20,22,27]. On the other hand, some p53-mediated miRNAs affect cellular outcome specifically. For example, miR-192 contributes to p53-dependent cell cycle arrest [21]. miR-34 facilitates apoptosis induction by repressing *SIRT1* or *Bcl-2* [28,29]. miR-22 promotes apoptosis by repressing *p21* [29]. Several models have been developed to illustrate the roles for p53-induced miRNAs in cell fate decision. Zhou et al. found that miR-605 can advance the induction of apoptosis by activating p53, while miR-34a promotes apoptosis by downregulating the antiapoptotic factors [30]. Issler et al. investigated the role of miR-16 in the conversion between cell senescence and apoptosis [31]. Moore et al. verified that p53-induced miR-192 ensures robust oscillations of p53 [32]. However, the coordination between p53-mediated proarrest and proapoptotic miRNAs has seldom been considered in modeling. Both miR-192 and miR-22 are induced by p53 in HCT116 cells [21]. It is feasible to select these two miRNAs as representatives to investigate how different p53-targeted miRNAs coordinate in cell fate decision.

We developed an integrated model of p53 signaling network to investigate the influence of miR-192 and miR-22 on p53 dynamics and decision-making between life and death in response to DNA damage. We found that p53 is activated in a progressive mode depending on the extent of DNA damage. For moderate damage, miR-192 promotes p53 accumulation to an intermediate level so that p21 is induced to trigger cell cycle arrest. Upon severe damage, p53 rises to rather high levels and transactivates miR-22, which represses *p21* expression and facilitates apoptosis induction. p53-induced phosphatase and tensin homolog (PTEN) is crucial for the full activation of p53 and apoptosis induction. Together, the level of active p53 affects the selective induction of its target genes, and p53-mediated proarrest and proapoptotic miRNAs are orchestrated in cell fate decision in the DDR.

## 2. Materials and Methods

### 2.1. Model

**Figure 1:**
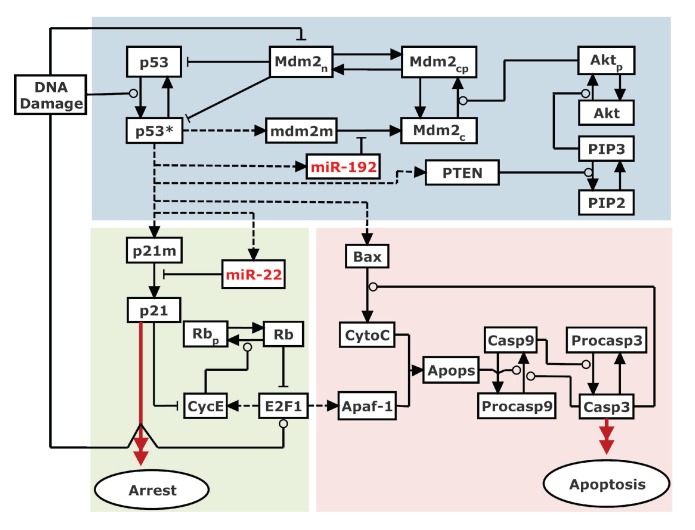
Schematic diagram of the integrated model. The model is mainly composed of three modules. The p53 regulation module considers three p53-centered feedback loops: one p53-Mdm2 negative feedback loop and two p53-PTEN-Akt-Mdm2 and p53-miR-192-Mdm2 positive feedback loops. Cell cycle arrest is induced through p21-dependent inhibition of E2F1, while apoptosis is triggered via the caspase cascade activated by Bax and Apaf-1. Notably, miR-22-dependent *p21* repression facilitates E2F1 activation. Dashed lines denote gene transcription induced by p53 and E2F1. Solid arrowed lines represent transitions between different forms. Promotion and inhibition of the components are denoted by circle-headed and bar-headed lines, respectively.

Experimentally, the roles of miR-22 and miR-192 in cell cycle arrest and apoptosis were investigated in p53 wild-type cells treated with adriamycin (ADR) and other DNA damaging agents [21,33]. Based on the experimental data, we built an integrated model of p53 signaling network to explore the roles of miR-192 and miR-22 in the DDR. This model is composed of three modules, separately characterizing p53 regulation, cell cycle control and apoptosis induction (Figure 1). Here, we focused on the modulation of p53 activation by miR-192 and regulation of apoptosis by miR-22 in response to ADR-induced DNA damage. Of note, we ignored DNA repair since DNA damage persistently exists in this case. The key points of the model are presented as follows.

### 2.2. Regulation of p53 Activity

p53-centered feedback loops are key players shaping its dynamics. Three feedback loops are considered here, i.e., the p53-Mdm2 negative feedback loop and the p53-miR-192-Mdm2 and p53-PTEN-Akt-Mdm2 positive feedback loops. We consider two forms of nuclear p53: p53 (nonphosphorylated, inactive) and p53^∗^ (phosphorylated, active). For simplicity, cytoplasmic p53 is not included in the model. Three forms of Mdm2 are considered: cytoplasmic dephosphorylated $\mathrm{Mdm}2_{c}$, cytoplasmic phosphorylated $\mathrm{Mdm}2_{\mathrm{cp}}$, and nuclear $\mathrm{Mdm}2_{n}$. The *mdm2* mRNA (mdm2m) is also included in our model. We assume that only $\mathrm{Mdm}2_{\mathrm{cp}}$ can enter the nucleus [34].

DNA damage activates p53 by phosphorylation that blocks p53 degradation by Mdm2 [35]. p53-induced miR-192 represses the synthesis of Mdm2 post-transcriptionally, thereby promoting p53 stabilization and enclosing a positive feedback loop [27]. The effect of miR-192 on Mdm2 production is described by inhibition of *mdm2* translation (see Equations (7) and (8) in Supporting Material). Moreover, p53-induced PTEN dephosphorylates phosphatidylinositol 3,4,5-trisphosphate (PIP3) and indirectly represses the phosphorylation of Akt, thereby preventing nuclear entry of $\mathrm{Mdm}2_{c}$ [34,36], enclosing another positive feedback loop.

The expression of each target gene by p53 is represented by a Hill function, and the Hill coefficient is set to 4 given that p53 functions as a transcription factor in a tetrameric form [37]. The total level of Akt is assumed to be constant since no remarkable changes were observed in the DDR [38]. Similarly, the total level of PIP3 and PIP2 is also assumed to be a constant.

### 2.3. Control of the Cell Cycle by the Rb/E2F1 Pathway

It is assumed that p53 promotes *p21* transcription [7], and p21 inhibits cyclin-dependent kinase 2 (Cdk2) by forming a complex with cyclin E (CycE) [9]. We assumed that both p21 and CycE can be degraded from the p21/CycE complex, which means as p21 is degraded, CycE is released and vice versa (Equation (21)).

p21-dependent inhibition of Cdk2 activity blocks the phosphorylation of Rb so that depophosphorylated Rb suppresses E2F1, leading to cell cycle arrest [39]. For simplicity, we consider either E2F1 or CycE as an indicator of cell cycle progression. There exists a positive feedback between CycE induction and E2F1 activation as E2F1 induces *CycE* to promote its own activation [40]. Therefore, p53-induced p21 can inhibit E2F1 to arrest the cell cycle in G1 phase.

The dynamic characterization of cell cycle regulation by the Rb/E2F1 pathway is derived from our previous model [41] (Equations (16)–(30)). p21 exists in two forms: p21 (free p21) and p21CE (p21 in complex with CycE). Three forms of Rb are considered: Rb (free non-phosphorylated Rb), Rbp (free hyperphosphorylated Rb), and RE (Rb in complex with E2F1). E2F1 exists in two forms: E2F1 (active free E2F1) and RE (inactive Rb-bound E2F1). The total levels of p21, Cyclin E, Rb and E2F1 are denoted by [$p21_{\mathrm{tot}}$], [${\mathrm{CycE}}_{\mathrm{tot}}$], [${\mathrm{Rb}}_{\mathrm{tot}}$] and [$E2F1_{\mathrm{tot}}$], respectively. [$p21_{\mathrm{tot}}$] and [${\mathrm{CycE}}_{\mathrm{tot}}$] are controlled by the levels of p53^∗^ and free E2F1, respectively [7,40]. E2F1 is assumed to be produced at a constant rate and be degraded at a DNA damage-dependent rate. [${\mathrm{Rb}}_{\mathrm{tot}}$] is assumed to be a constant.

### 2.4. Apoptosis Induction

p53-induced Bax plays a significant role in apoptosis induction [8]. It forms oligomers to trigger mitochondrial outer membrane permeabilization (MOMP), leading to the release of cytochrome *c* (CytoC) into the cytoplasm [42]. For simplicity, the inhibitors of apoptosis like Bcl-xL are not included in the current model [43]. In addition, E2F1 is considered to facilitate cell death by inducing *Apaf-1* [44]. CytoC and Apaf-1 can form apoptosome (Apops), which recruits procaspase-9 (Procasp9) and activates it into caspase-9 (Casp9). Casp9 further activates procaspase-3 (Procasp3) into caspase-3 (Casp3) and apoptosis ensues [45]. Furthermore, Casp3 also amplifies the activation of caspase-9 and CytoC release by cleaving its inhibitors [46,47]. The dynamics of this module are characterized by Equations (31)–(36).

Notably, p21 is assumed to antagonize apoptosis by suppressing Apaf-1 expression via inhibiting E2F1, based on a previous report that p21 impedes apoptosis by repression of CDK-dependent Casp9 activation [48]. In contrast, p53-inducible miR-22 can promote apoptosis by post-transcriptionally repressing *p21* expression in the DDR [33]. We also assume that the thresholds for transactivating p53-inductive proapoptotic genes (like *PTEN*, *miR-22* and *Bax*) are higher than those for the proarrest genes (like p21 and miR-192), in accordance with the experimental report [49].

### 2.5. Methods

The concentration of each species is symbolized by a variable in rate equations in Supplemental Method S1 in Supporting Material. The reactions related to phosphorylation and dephosphorylation are characterized by Michaelis–Menten kinetics. The description of variables and their initial values are listed in Supplementary Table S1. The standard parameter values are listed in Supplemental Table S2. Time is in units of minutes, and the intensity of DNA damage is denoted by DD. The units of parameters are determined such that the concentration of proteins is dimensionless. We solved the differential equations numerically and plotted the bifurcation diagrams by the free software Oscill8 v2.0 (available online: http://oscill8.sourceforge.net/).

## 3. Results

### 3.1. Dynamics of the Key Network Components in the DDR

**Figure 2:**
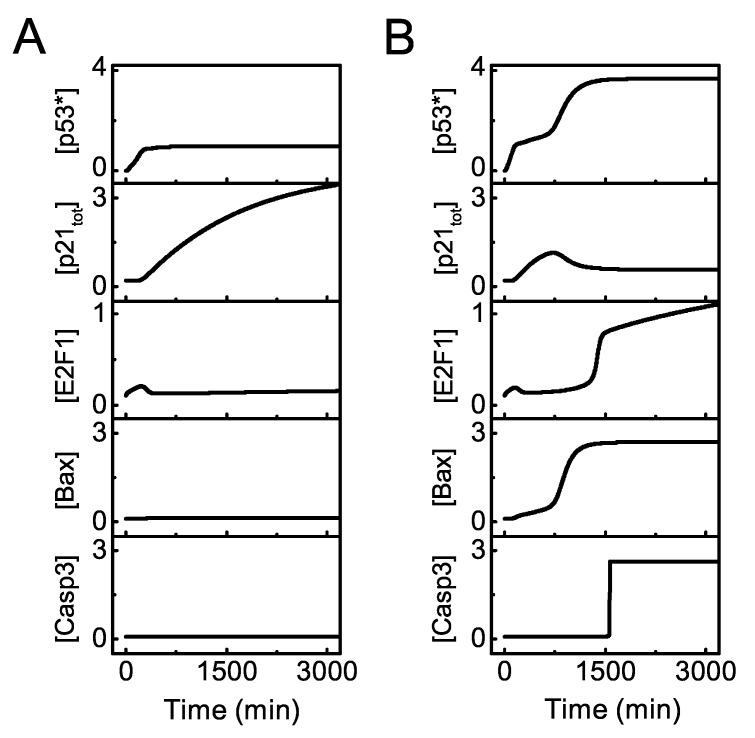
Overview of the dynamics of p53 signaling network. Temporal evolution of [$p53^{*}$], [$p21_{\mathrm{tot}}$], [E2F1], [Bax], and [Casp3] (from top to bottom) with DD = 25 (**A**) or 100 (**B**).

We first display the dynamics of the key components of the signaling network for distinct intensities of DNA damage. For moderate damage (DD=25), p53^∗^ rises to an intermediate level (Figure 2A). p21 is induced to repress E2F1 activation, while Bax and Casp3 remain at low levels, leading to cell cycle arrest. For severe damage (DD=100), the system shows two-phase dynamics (Figure 2B). [p53^∗^] subsequently reaches moderate and high levels. p21 rises transiently and then drops to rather low levels. After a time delay, E2F1 is activated, Bax accumulates remarkably and Casp3 is triggered in a switch-like manner. Thus, the cell undergoes transient cell cycle arrest and commits suicide finally in response to severe damage.

**Figure 3:**
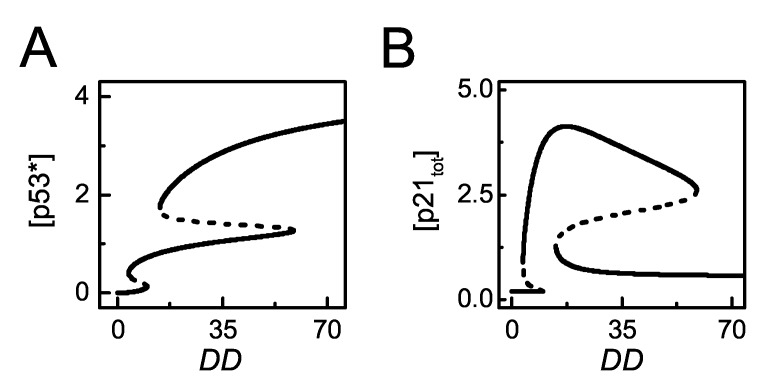
The steady-state behaviors of [$p53^{*}$] and [$p21_{\mathrm{tot}}$]. (**A**,**B**) Bifurcation diagram of [p53^∗^] (**A**) or [p21_tot_] (**B**) versus DD. The stable and unstable steady states are indicated by solid and dashed lines, respectively (the same below).

The bifurcation diagrams of [p53^∗^] and [p21_tot_] versus *DD* are plotted to show the dependency of p53 activation and p21 expression on the severity of DNA damage (Figure 3). p53 exhibits two bistable switches with increasing damage. There exist two thresholds of *DD* during the progressive activation of p53. For slight damage, p53 remains inactive. For moderate damage, it is partially activated. For severe damage, it is fully activated (Figure 3A). p21 rises to high levels and stays at rather high levels upon moderate damage, while it drops to basal levels for severe damage (Figure 3B). Together, p53 is gradually activated in the DDR, while p21 levels vary non-monotonically with increasing DNA damage [33].

### 3.2. The Role of miR-192 in Cell Cycle Arrest

**Figure 4:**
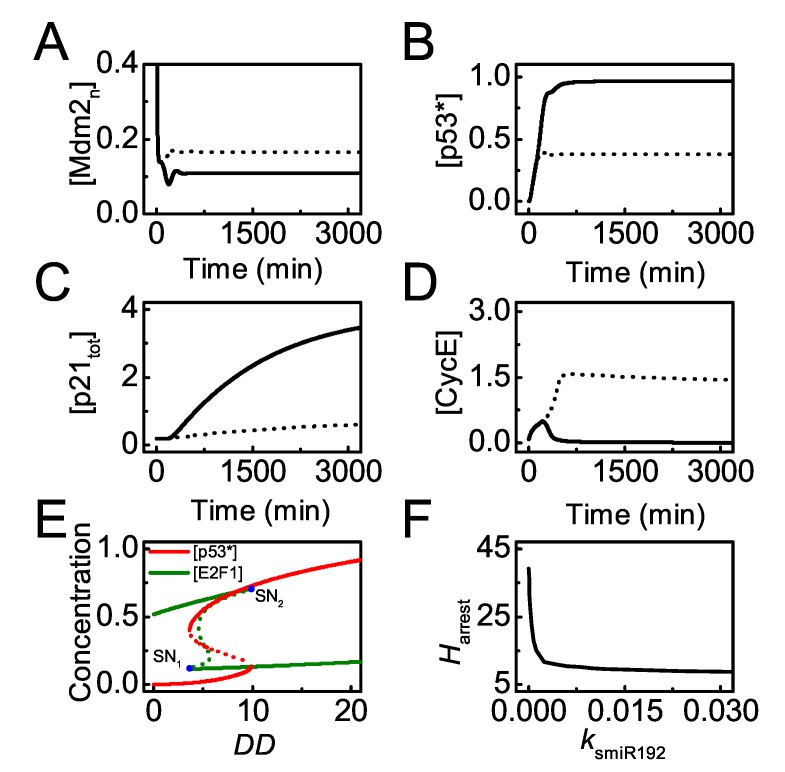
miR-192 promotes cell cycle arrest. (**A**–**D**) Time courses of [$\mathrm{Mdm}2_{n}$] (**A**), [$p53^{*}$] in (**B**), [$p21_{\mathrm{tot}}$] (**C**) and [CycE] (**D**) with the p53-induced synthesis rate of miR-192, $k_{\mathrm{smiR}192}$ = 0 (dash) and 0.01 (solid, the standard case) with DD = 25; (**E**) bifurcation diagrams of [$p53^{*}$] (red) and [E2F1] (dark green) versus DD. Blue dots mark saddle-node bifurcations (${\mathrm{SN}}_{1}$, ${\mathrm{SN}}_{2}$), and the value of DD at ${\mathrm{SN}}_{2}$ refers to the threshold for cell cycle arrest, $H_{\mathrm{arrest}}$. (**F**) The curve of $H_{\mathrm{arrest}}$ versus $k_{\mathrm{smiR}192}$.

miR-192 plays a primary role in cell cycle progression [21,27], and it is worth studying how miR-192 influences cell proliferation. When miR-192 is normally induced by p53 upon mild damage (*DD* = 25), p53 reaches a moderate level due to little Mdm2 in the nucleus, and p21 is induced to arrest the cell cycle by repressing *CycE* (see the solid lines in Figure 4A–D). By contrast, in the absence of miR-192 induction ($k_{\mathrm{smiR}192}$ = 0), the steady state of [$\mathrm{Mdm}2_{n}$] rises markedly and [p53^∗^] drops remarkably due to degradation by Mdm2 (Figure 4A,B, dotted lines). p21 expression is repressed significantly and CycE rises to high levels promoting normal proliferation (Figure 4C,D, dotted lines). Therefore, miR-192 contributes to p21-dependent cell cycle arrest by stabilizing p53 indirectly, consistent with the experimental results [27].

We have illustrated that p53-induced *miR-192* expression is required for cell cycle arrest upon mild damage. To show the significance of miR-192 in cell cycle arrest globally, we further analyze how miR-192 level modulates the threshold of *DD* for cell cycle arrest. Figure 4E shows the bifurcation diagrams of [p53^∗^] and [E2F1] versus *DD* with the standard parameter setting. Only when *DD* exceeds its higher threshold does p53^∗^ switch to moderate levels while E2F1 drops to low levels. This threshold is considered the threshold of *DD* for cell cycle arrest, denoted by $H_{\mathrm{arrest}}$. Furthermore, the dependency of $H_{\mathrm{arrest}}$ on the induction rate of miR-192 is described by the bifurcation diagram of $H_{\mathrm{arrest}}$ versus $k_{\mathrm{smiR}192}$ (Figure 4F). $H_{\mathrm{arrest}}$ decreases monotonically with increasing $k_{\mathrm{smiR}192}$, meaning that cells with more miR-192 can exit from cell cycle for milder damage. Together, p53-induced miR-192 acts as a key modulator of cell cycle arrest.

### 3.3. The Role of miR-22 in Apoptosis

**Figure 5:**
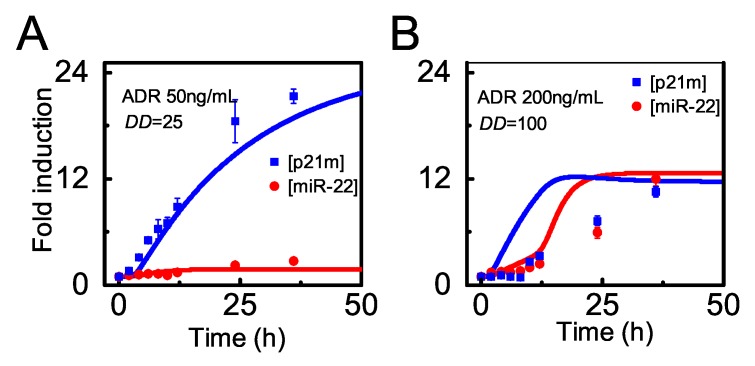
Time courses of *p21* mRNA and miR-22 under two distinct conditions. Time courses of [p21m] (*p21* mRNA, blue) and [miR-22] (red) upon moderate (**A**) or severe (**B**) damage based on the simulation results (line) or normalized experimental data (dot) retrieved from Ref. [33].

It has been verified that *miR-22* is induced by p53 and represses *p21* expression in HCT116 cells treated with lethal dose of ADR [33]. First, we compare the simulation results with the experimental data from cells treated with 50 or 200 ng/mL ADR after normalization (Figure 5). Upon sublethal damage, miR-22 remains at rather low levels while *p21* mRNA rises to high levels without marked repression from miR-22. The simulation results at DD=25 agree well with the experimental data for 50 ng/mL ADR treatment [33] (Figure 5A). Upon lethal damage, miR-22 rises to high levels so that *p21* mRNA is partially degraded and only rises to moderate levels (Figure 5B). In this case, the time courses of miR-22 are fairly consistent with most experimental data except the data point at 24 h. There exists some deviation from experimental data for *p21* mRNA during the rising phase. Of note, both miR-22 and *p21* mRNA rise more rapidly in the simulation results since the time delay in p53-induced target gene expression is not considered in our model.

**Figure 6:**
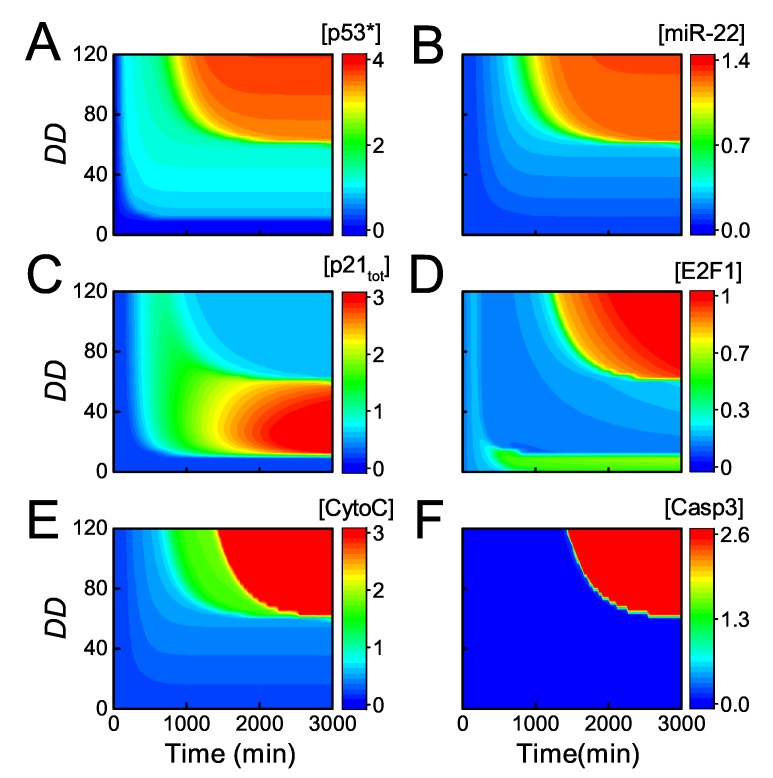
Dynamics of the key network components for varying intensities of DNA damage. The heatmap of [$p53^{*}$] (**A**); [miR-22] (**B**); [$p21_{\mathrm{tot}}$] (**C**); [E2F1] (**D**); [CytoC] (**E**); and [Casp3] (**F**) as a function of Time and DD.

It has been reported that *p21* induction results in cell-cycle arrest in cells treated by 50 ng/mL ADR, while inhibition of *p21* induction by miR-22 leads to apoptosis upon lethal dose of ADR [33]. Next, we show the signaling process of cell fate determination globally by the heat map of the key components as functions of DD and Time (Figure 6). For slight damage (DD < 10), p53 and p21 are inactivated, and E2F1 keeps active so that cells proliferate normally (Figure 6A–D). For mild or moderate damage (10 < DD < 60), p53 is primarily activated while miR-22 is still not induced since the threshold of p53 for its transactivation is rather high [33] (Figure 6A,B). Consequently, p21 is highly expressed resulting in repression of E2F1, leading to cell cycle arrest (Figure 6C,D).

Upon severe damage (DD > 60), the system undergoes two-phase dynamics. In the first phase, [$p53^{*}$] reaches an intermediate level and p21 rises to moderate levels, resulting in transient cell cycle arrest (Figure 6A–C). In the second phase, [$p53^{*}$] is fully activated and still induces both p21 and miR-22, but p21 protein drops to rather low levels owing to post-transcriptional repression by miR-22 (Figure 6A–C). Therefore, E2F1 rises to high levels finally and induces Apaf-1 (Figure 6D, see also Supplemental Figure S1). At the same time, fully active p53 induces Bax, resulting in the release of CytoC (Figure 6E, see also Supplemental Figure S1). As a result, CytoC and Apaf-1 assemble into apoptosome (Apops) to provide a platform for activation of Casp9, and Casp3 is further activated to trigger apoptosis (Figure 6F). Of note, both high expression of Bax and Apaf-1 is required for activating the caspase cascade for apoptosis induction. Taken together, our model is able to reveal the regulation of cell fate decision by p53-induced miR-22 and p21.

### 3.4. Regulation of miR-22 and p21 in Apoptosis Induction

**Figure 7:**
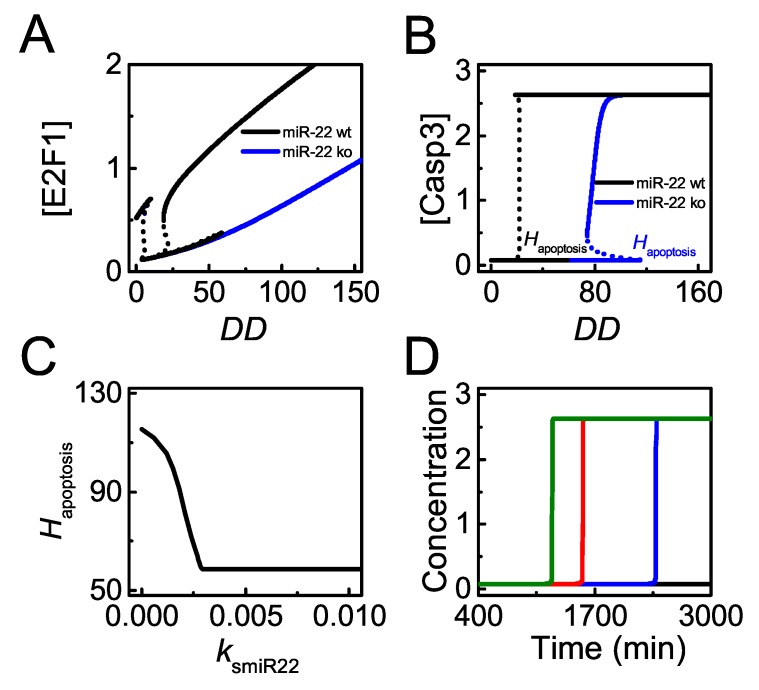
miR-22 sensitizes cells to p53-dependent apoptosis induced by DNA damage. (**A**,**B**) bifurcation diagram of [E2F1] (**A**) or [Casp3] (**B**) versus DD with the p53-induced synthesis rate of miR-22, $k_{\mathrm{smiR}22}$ = 0.0075 (black, the default case) and 0 (blue), respectively. The threshold of DD for apoptosis is denoted by $H_{\mathrm{apoptosis}}$; (**C**) the curve of $H_{\mathrm{apoptosis}}$ versus $k_{\mathrm{smiR}22}$; (**D**) time courses of [Casp3] with $k_{\mathrm{smiR}22}$ = 0.015 (green), $k_{\mathrm{smiR}22}$ = 0.0075 (red, the default case), $k_{\mathrm{smiR}22}$ = 0.0045 (blue), and $k_{\mathrm{smiR}22}$ = 0.0015 (black) at DD = 100.

As clarified above, miR-22 promotes p53-dependent apoptosis by repressing *p21* expression upon severe damage. Next, we will explore how miR-22 abundance influences apoptosis induction. Figure 7A,B show the bifurcation diagrams of [E2F1] and [Casp3] versus DD with wild-type *miR-22* or knockout. In normal case, E2F1 stays at moderate levels in proliferating cells upon slight damage. When DNA damage is moderate, E2F1 drops to low levels corresponding to cell cycle arrest due to p21 induction (Figure 7A). When DNA damage increases further and exceeds the threshold for apoptosis induction, $H_{\mathrm{apoptosis}}$, p53 induces *miR-22* to repress *p21* expression, leading to E2F1 reactivation (Figure 7A, see also Figure 6). As a result, Casp3 is activated to initiate apoptosis upon lethal damage (Figure 7B). When *miR-2*2 is knocked out, p21 is always highly expressed upon DNA damage. Considering the stabilization of E2F1 by DNA damage [50], E2F1 can gradually rise to moderate levels with increasing DD even without miR-22 induction (Figure 7A). Consequently, E2F1 induces apoptosis by activating Casp3 only for very severe damage in *miR-22* knockout case (Figure 7B). Therefore, cells with *miR-22* knockout become more resistant to DNA damage.

The curve of $H_{\mathrm{apoptosis}}$ versus $k_{\mathrm{smiR}22}$ (p53-dependent induction rate of miR-22) can illuminate the role of miR-22 in apoptosis more globally (Figure 7C). $H_{\mathrm{apoptosis}}$ decreases with increasing $k_{\mathrm{smiR}22}$ and reaches a fixed value for larger $k_{\mathrm{smiR}22}$ (Figure 7C). Of note, p53 and E2F1 should be activated to separately induce *Bax* and *Apaf-1* in apoptosis induction (see also Supplemental Figure S1). Therefore, $H_{\mathrm{apoptosis}}$ is defined by the higher one between the thresholds of DD for activating the two transcription factors. For smaller $k_{\mathrm{smiR}22}$, E2F1 rises to sufficiently high levels when DD exceeds a rather high threshold due to low levels of miR-22. In this case, $H_{\mathrm{apoptosis}}$ is determined by the threshold of DD for E2F1 reactivation and it decreases with increasing $k_{\mathrm{smiR}22}$. For larger $k_{\mathrm{smiR}22}$, the threshold of DD for p53 full activation is not lower than that for E2F1 full activation so that $H_{\mathrm{apoptosis}}$ takes the former threshold which is independent of $k_{\mathrm{smiR}22}$.

Upon severe damage (DD = 100), the time required for Casp3 activation increases with decreasing $k_{\mathrm{smiR}22}$, and apoptosis does not occur for rather small $k_{\mathrm{smiR}22}$ (Figure 7*D*). These illustrate that high miR-22 expression sensitizes cells to p53-dependent apoptosis, consistent with the experimental result that transfection of miR-22 caused a marked increase of apoptotic cells treated by ADR [33]. Moreover, cellular outcome will transform from apoptosis to cell cycle arrest because of p21 upregulation when $k_{\mathrm{smiR}22}$ decreases markedly upon severe damage, showing agreements with experimental data that miR-22 knockout inhibits apoptosis in HCT116 cells treated by lethal dose of ADR [33].

**Figure 8:**
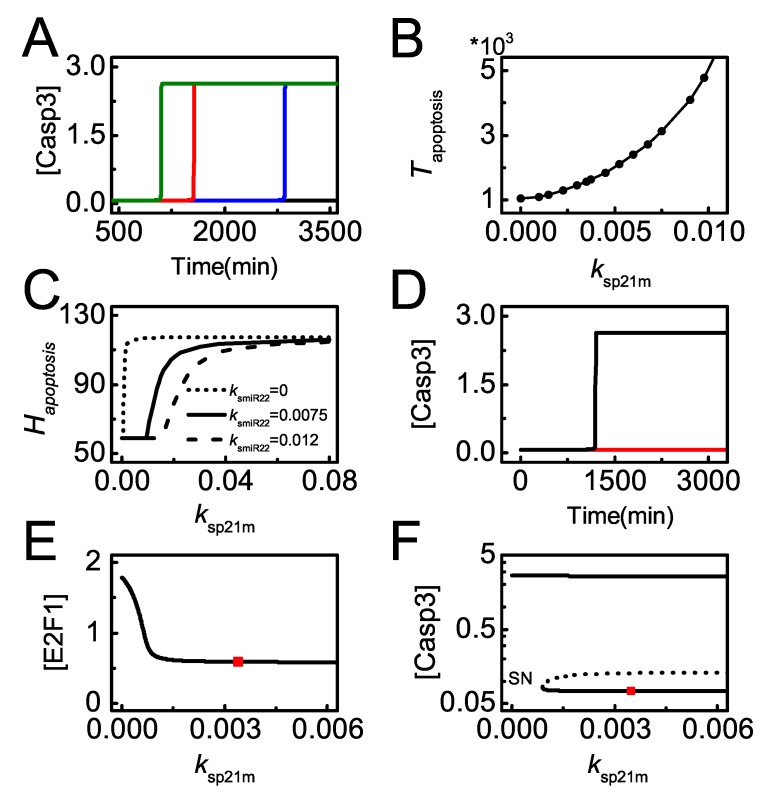
p21 downregulation promotes miR-22-dependent apoptosis. (**A**) Time courses of [Casp3] with DD = 100 and $k_{\mathrm{sp}21m}$ = 0.001 (green), 0.0035 (red, the default case), 0.007 (blue) or 0.018 (black); (**B**) the curve of $T_{\mathrm{apoptosis}}$ (time required for Casp3 activation) versus $k_{\mathrm{smiR}22}$; (**C**) the curves of $H_{\mathrm{apoptosis}}$ versus $k_{\mathrm{sp}21m}$ for different $k_{\mathrm{smiR}22}$; (**D**) time courses of [Casp3] for DD = 100 with $k_{\mathrm{sp}21m}$ = 0.0003 (black) or 0.0035 (red, the default case) in the case of miR-22 knockout; (**E**,**F**) Bifurcation diagram of [E2F1] and [Casp3] versus $k_{\mathrm{sp}21m}$ with $k_{\mathrm{smiR}22}$ = 0 and DD = 100. $k_{\mathrm{sp}21m}$ takes the default value at the position marked by the red square. “SN” denotes saddle-node bifurcation point.

We further analyze the effect of *p21* transcription on apoptosis induction. In severely damaged cells, p21 transcription rate significantly influences the timing of apoptosis. The required time for apoptosis increases with increasing $k_{\mathrm{sp}21m}$ (p53-dependent *p21* transcription rate) and even apoptosis can’t be initiated with rather large $k_{\mathrm{sp}21m}$ (Figure 8A). Thus, enhanced *p21* transcription counteracts the repression of its translation from miR-22 and prevents cells from apoptosis even for lethal damage. The timing of Casp3 activation is defined as the time required for apoptosis, $T_{\mathrm{apoptosis}}$. It rises continuously when $k_{\mathrm{sp}21m}$ increases from 0 to 0.01 at DD=100 (Figure 8B). These indicate that *p21* overexpression can attenuate miR-22-stimulated apoptosis, while p21 deficiency sensitizes cells to apoptosis upon severe damage [51].

The antagonism between miR-22 and p21 in apoptosis is more clearly exhibited by the curve of $H_{\mathrm{apoptosis}}$ versus $k_{\mathrm{sp}21m}$ (Figure 8C). $H_{\mathrm{apoptosis}}$ looks like a monotonically-increasing sigmoid function of $k_{\mathrm{sp}21m}$. The curve shifts rightward with increasing $k_{\mathrm{smiR}22}$, meaning that $H_{\mathrm{apoptosis}}$ decreases with increasing $k_{\mathrm{smiR}22}$. Moreover, in *miR-22* knockout cells, Casp3 remains inactive when $k_{\mathrm{sp}21m}$ takes the default value, while Casp3 is activated when $k_{\mathrm{sp}21m}$ reduces remarkably (Figure 8D). This suggests that miR-22-deficient cells can be killed through repressing *p21* transcription. In the *miR-22* knockout case, the bifurcation diagrams of [E2F1] versus $k_{\mathrm{sp}21m}$ shows that the steady-state level of E2F1 drops to rather low levels when $k_{\mathrm{sp}21m}$ exceeds a certain threshold (Figure 8E). Our result suggests that p21 promotes cell survival by repressing E2F1 that promotes apoptosis by inducing *Apaf*-1 [52]. Correspondingly, Casp3 stays at low levels when $k_{\mathrm{sp}21m}$ is greater than the threshold, and it is activated only when $k_{\mathrm{sp}21m}$ becomes small enough (Figure 8F). Together, it is a potent mechanism for miR-22 to modulate apoptosis by repressing *p21* expression.

### 3.5. Role of miR-192 and PTEN in p53-Dependent Apoptosis

We have shown that p53-induced miR-22 promotes apoptosis by repressing *p21* expression post-transcriptionally. It has been identified that the full activation of p53 is required for cell death in response to lethal damage [53]. miR-192 promotes p53 activation by repressing *Mdm2* expression [27], while PTEN contributes to p53 stabilization by blocking the nuclear entry of Mdm2 [36], enclosing two p53-centered positive feedback loops. Next, we will investigate how miR-192 and PTEN contribute to p53-dependent apoptosis.

**Figure 9:**
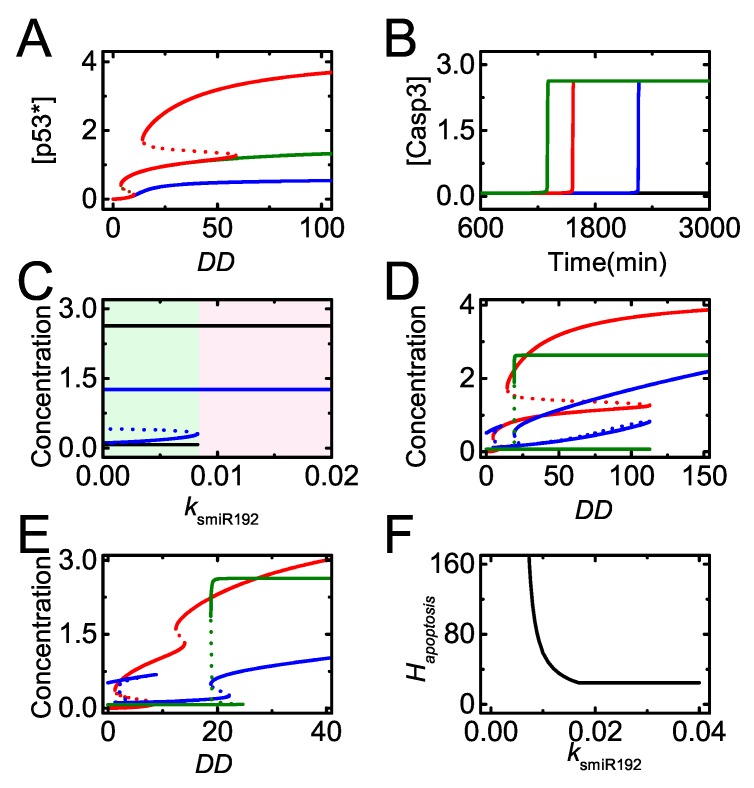
The induction of miR-192 is required for apoptosis induction. (**A**) Bifurcation diagram of [$p53^{*}$] versus DD for $k_{\mathrm{smiR}192}$ = 0, $k_{\mathrm{spten}}$ = 0 or 0.06 (blue, the lines for the two cases are overlapped), $k_{\mathrm{smiR}192}$ = 0.01, $k_{\mathrm{spten}}$ = 0 (green), and $k_{\mathrm{smiR}192}$ = 0.01, $k_{\mathrm{spten}}$ = 0.06 (red); (**B**) time courses of [Casp3] with $k_{\mathrm{smiR}192}$ = 0.02 (dark green), 0.01 (red, the default case), 0.0085 (blue) and 0.006 (black) at DD = 100; (**C**) bifurcation diagrams of [miR-22] (blue) and [Casp3] (black) versus $k_{\mathrm{smiR}192}$ with DD = 100. Here, $k_{\mathrm{smiR}192}$ varies from 0 to twice the default value. For relatively small $k_{\mathrm{smiR}192}$, cells will undergo cell cycle arrest (light green region), while for rather large $k_{\mathrm{smiR}192}$, cells will undergo apoptosis (light pink region). (**D**,**E**) Bifurcation diagrams of [p53^∗^], [E2F1] and [Casp3] versus DD with $k_{\mathrm{smiR}192}$ = 0.008 (**D**) and 0.03 (**E**); (**F**) the curve of $H_{\mathrm{apoptosis}}$ versus $k_{\mathrm{smiR}192}$.

The bifurcation diagram of [$p53^{*}$] versus DD is plotted for different miR-192 and PTEN expression states (Figure 9A). In the normal case, $p53^{*}$ reaches intermediate levels upon moderate damage and further rises to high levels upon severe damage (see the red line). Once *miR-192* is knocked out, [$p53^{*}$] always stays at low levels (see the blue line). When *PTEN* alone is knocked out, [$p53^{*}$] only reaches an intermediate level (see the green line). Our results indicate that miR-192 is required for both the primary and full activation of p53, while PTEN only contributes to the full activation of p53. The effect of *miR-192* expression on apoptosis is described by the dynamics of [Casp3] with different $k_{\mathrm{smiR}192}$ (Figure 9B). The time required for Casp3 activation increases with decreasing $k_{\mathrm{smiR}192}$, and apoptosis cannot be induced with rather small $k_{\mathrm{smiR}192}$. Therefore, sufficient miR-192 is required for p53 activation and apoptosis induction.

We further explore the influence of miR-192 induction rate on apoptosis induction by the bifurcation diagrams of [miR-22] and [Casp3] versus $k_{\mathrm{smiR}192}$ for severe damage (Figure 9C). When $k_{\mathrm{smiR}192}$ is below the threshold, both [miR-22] and [Casp3] stay at their low states and apoptosis will not occur in severely damaged cells. With sufficient miR-192, miR-22 reaches its high state and apoptosis is triggered by activated Casp3. Thus, miR-192 abundance modulates the decision between cell cycle arrest and apoptosis by regulating miR-22 expression and Casp3 activation indirectly.

In our model, apoptosis needs both *Bax* and *Apaf-1* induction by fully activated p53 and E2F1, respectively, so that the threshold of apoptosis is determined by the higher one among the two DD thresholds for p53 and E2F1 activation. We plot the bifurcation diagrams of [p53^∗^], [E2F1] and [Casp3] versus DD with smaller or larger $k_{\mathrm{smiR}192}$ (Figure 9D,E). For smaller $k_{\mathrm{smiR}192}$, rather severe damage is required to fully activate p53 and the threshold of DD for apoptosis is determined by p53 activation (Figure 9D). By contrast, for large $k_{\mathrm{smiR}192}$, even upon moderate damage, p53 can be activated, so that the threshold of DD for E2F1 to induce *Apaf-1* defines $H_{\mathrm{apoptosis}}$ (Figure 9E).

We further reveal the influence of $k_{\mathrm{smiR}192}$ on apoptosis induction globally by the curve of $H_{\mathrm{apoptosis}}$ versus $k_{\mathrm{smiR}192}$ (Figure 9F). For rather small $k_{\mathrm{smiR}192}$, apoptosis can hardly be induced. $H_{\mathrm{apoptosis}}$ decreases quickly with increasing $k_{\mathrm{smiR}192}$ when it takes medium values. When $k_{\mathrm{smiR}192}$ exceeds some threshold, $H_{\mathrm{apoptosis}}$ will not vary with increasing $k_{\mathrm{smiR}192}$ and fix at a constant. Theses results can be explained by the roles of p53 and E2F1 pathways in apoptosis induction. When $k_{\mathrm{smiR}192}$ is small, the threshold of DD for p53 full activation defines $H_{\mathrm{apoptosis}}$, which decreases with increasing $k_{\mathrm{smiR}192}$ (see also Figure 9D). By contrast, for large $k_{\mathrm{smiR}192}$, the threshold of DD for E2F1-dependent Apaf-1 induction defines $H_{\mathrm{apoptosis}}$ (see also Figure 9E). The repression of *p21* by miR-22 becomes saturated with high miR-192 expression so that the activation of DD is independent of $k_{\mathrm{smiR}192}$ and $H_{\mathrm{apoptosis}}$ keeps a constant with increasing $k_{\mathrm{smiR}192}$. Therefore, miR-192 promotes p53 activation and modulates E2F1 activation through p53-induced miR-22, thereby influencing apoptosis induction.

**Figure 10:**
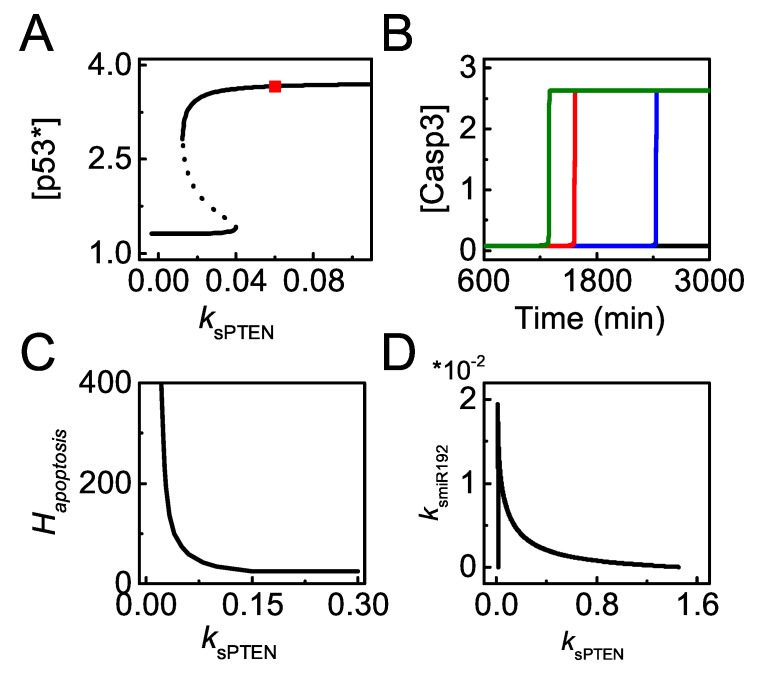
Effect of PTEN induction on p53 activity and cellular outcome. (**A**) bifurcation diagram of [$p53^{*}$] versus $k_{\mathrm{sPTEN}}$ with DD = 100. The red square symbolizes the default case. (**B**) time courses of [Casp3] with $k_{\mathrm{sPTEN}}$ = 0.2 (dark green), 0.06 (red, the default case), 0.042 (blue), and 0.03 (black) at DD = 100; (**C**) the curve of $H_{\mathrm{apoptosis}}$ versus $k_{\mathrm{sPTEN}}$; (**D**) the two-parameter bifurcation diagram of $k_{\mathrm{smiR}192}$ versus $k_{\mathrm{sPTEN}}$.

The effects of *PTEN* induction on p53-dependent apoptosis are further investigated. The bifurcation diagram of [$p53^{*}$] versus $k_{\mathrm{sPTEN}}$ shows that the steady states of [$p53^{*}$] exhibit bistability with varying $k_{\mathrm{sPTEN}}$ upon severe damage (DD = 100). $p53^{*}$ settles at moderate levels when $k_{\mathrm{sPTEN}}$ is below the threshold; it rises to high levels and is fully activated when $k_{\mathrm{sPTEN}}$ exceeds the threshold (Figure 10A). Moreover, the time required for Casp3 activation increases with decreasing $k_{\mathrm{sPTEN}}$, and apoptosis cannot take place in PTEN-deficient cells (Figure 10B). Taken together, sufficient PTEN is required for the full activation of p53 and apoptosis induction in severely damaged cells.

$H_{\mathrm{apoptosis}}$ decreases with increasing $k_{\mathrm{sPTEN}}$ and reaches a constant value with large $k_{\mathrm{sPTEN}}$ (Figure 10C). When $k_{\mathrm{sPTEN}}$ is very large, p53 can be fully activated with rather small DD, and [$p53^{*}$] becomes saturated with further increasing $k_{\mathrm{sPTEN}}$. As a result, p21 almost keeps constant with increasing $k_{\mathrm{sPTEN}}$ and the threshold of DD for E2F1 activation is independent of $k_{\mathrm{sPTEN}}$, and thereby $H_{\mathrm{apoptosis}}$ is a constant with greater $k_{\mathrm{sPTEN}}$. In a word, saturation of $H_{\mathrm{apoptosis}}$ with increasing $k_{\mathrm{smiR}22}$ results from the limit of p53 activation, while saturation in the $H_{\mathrm{apoptosis}}$ versus $k_{\mathrm{sPTEN}}$ curve roots in the limit of E2F1 reactivation upon DNA damage. Our results suggest that it is difficult to induce apoptosis when PTEN is deficient.

Given that both miR-192 and PTEN are amplifiers of p53 activation and contribute to apoptosis, it is necessary to explore whether miR-192 and PTEN interplay in facilitating apoptosis in response to lethal damage. We plot the two-parameter bifurcation diagram of $k_{\mathrm{smiR}192}$ versus $k_{\mathrm{sPTEN}}$ for severe damage (Figure 10D). The diagram characterizes the bifurcation points of $k_{\mathrm{sPTEN}}$ with varying $k_{\mathrm{smiR}192}$ corresponding to Figure 10A. The two lines define two regions in the parameter plane: the triangle region below the two lines corresponds to cell survival; the top-right region corresponds to apoptosis. miR-192 and PTEN compensate for each other in apoptosis induction, i.e., more PTEN is required to ensure apoptosis induction in miR-192 deficient cells, and vice versa. This compensation effect may result from the sharing roles of miR-192 and PTEN in repressing *Mdm2* [27,36]. Therefore, miR-192 and PTEN cooperate in apoptosis induction by promoting p53 activation.

## 4. Discussion

miR-192 mainly promotes cell cycle arrest in a subset of cancer cell lines such as HCT116 and U2OS [21,54]. Our model also reveals its function in promoting apoptosis. We propose that miR-192 may act as a pre-activator of p53 and facilitates the full activation of p53 by downregulating Mdm2. Only when p53 is primarily activated by miR-192 does it gradually induce *PTEN* to fully activate itself, initiating apoptosis upon lethal damage. The pro-apoptotic function of miR-192 is supported experimentally in which the apoptosis is markedly enhanced in MM1s cells with ectopic expression of miR-192 [27]. By contrast, *miR-192* knockdown significantly blocks vancomycin-induced apoptosis and caspase activity in HK-2 cells [55]. Moreover, antagonizing *miR-192-5p/215* expression attenuates curcumin-induced apoptosis in H460, A427 and A549 cells [56]. Our results also suggest that apoptosis may be induced in miR-192 overexpressed cells upon moderate damage. Therefore, more attention should be paid to the proapoptotic function of miR-192 in the future.

Our model focuses on the functions of miR-192 and miR-22 in cell fate decision. As illustrated above, miR-192 and miR-22 are frequently downregulated in many tumors compared with the normal tissues [21,33]. In our work, downregulation of miR-192 or miR-22 remarkably increases the threshold of DNA damage required for cell cycle arrest or apoptosis. It has been reported that transfection of *miR-192* augments the percentage of cell cycle arrest in Camptothecin-treated HCT116 cells while knockdown of miR-192 notably reduces cell arrest in Adriamycin-treated IMR90 cells [21,57]. In addition, low expression of miR-22 leads to reduction in apoptosis in Adriamycin-treated HCT116 cells compared with the case of miR-22 over-expression [33]. Our results suggest that their downregulation might confer a chance of cell survival with tumorigenic potential; in cancer cells, miR-192 or miR-22 knockdown makes them more resistant to genotoxic therapies. Therefore, elevating miR-192 or miR-22 expression would be a promising strategy for cancer therapy.

Coordination of p53 and E2F1 in apoptosis induction has been studied in both experiments and modeling. E2F1 activates p53 by inducing ARF in response to oncogene activation [58]. It can also upregulate p53DINP1 and ASPP to activate the proapoptotic activities of p53 in response to DNA damage [59]. Moreover, E2F1 induces *Apaf-1* and cooperates with the p53-dependent mitochondrial pathway to activate the caspase cascade [52,60]. Based on the above experimental results, we and others have developed models to investigate the cooperation of p53 and E2F1 pathway in apoptosis induction [24,41,61]. In the current work, we proposed that the threshold of DNA damage for apoptosis induction may be determined by either p53 or E2F1 pathway under different conditions. This interesting prediction could be verified by experiments in the future.

Our model emphasizes the significance of the miR-22-p21 axis in facilitating apoptosis induction in severely damaged cells. For severe damage, fully activated p53 transactivates *miR-22* to repress p53-targeted *p21* post-transcriptionally, thereby promoting apoptosis. Of note, p53, p21 and miR-22 form an incoherent feedforward loop, and it is intriguing to investigate the significance of such a characteristic motif in the p53 network [62]. Moreover, according to our work, the choice between cell cycle arrest and apoptosis can be mediated by controlling the relative abundance of miR-22 and p21. Knocking down *miR-22* may transform cellular phenotype from apoptosis to cell cycle arrest upon lethal damage, consistent with the experimental data [33]. Nevertheless, apoptosis can reoccur by reducing the production of p21 in cells with *miR-22* knockdown. p53-induced p21 and miR-22 compete to determine cell fate between survival and death in severely damaged cells. We expect that our work may provide cues for designing novel cancer treatment strategies.

## 5. Conclusions

We have developed a network model to probe how p53-inducible *miR-192* and *miR-2*2 coordinate to regulate the DDR. Our results indicate that miR-192 and miR-22 are induced under different conditions. Upon mild or moderate damage, *miR-192* is induced and contributes to the partial activation of p53, elevating *p21* expression and leading to cell cycle arrest; upon severe damage, p53 is fully activated and transactivates *miR-22* to repress *p21* expression at the post-transcriptional level and to trigger apoptosis. Of note, miR-192 and PTEN also cooperate in p53-dependent apoptosis. In summary, p53-induced miR-192 and miR-22 coordinate in the upstream and downstream of the network respectively to modulate cell fate decision.

## Supplementary Materials

The following are available online at https://www.mdpi.com/1422-0067/20/19/4768/s1, Figure S1: Heat map of [Bax] (*A*) and [Apaf-1] (*B*) as a function of Time and DD, Table S1: Variables and their initial values, Table S2: Parameter Values.

## Abbreviations

The following abbreviations are used in this manuscript:

DDR	DNA damage response
microRNA	miRNA
PTEN	Phosphatase and tensin homolog
MOMP	Mitochondrial outer membrane permeabilization
