# Supplementary material for: Coordination of miR-192 and miR-22 in p53-Mediated Cell Fate Decision
Source: Int J Mol Sci. 2019 Sep 26;20(19):4768. doi: 10.3390/ijms20194768 (PMC6801623; doi:10.3390/ijms20194768)
Supplement: Supplementary file 1 [file ijms-20-04768-s001.pdf]

# SUPPORTING MATERIAL

## Coordination of miR-192 and miR-22 in p53-mediated cell fate decision

Here, we present the ordinary differential equations for the model and standard parameter values and the initial values of variables .

### Supplemental Method S1: Ordinary Differential Equations for the Model

$$\frac{d[\text{p53}^*]}{dt} = k_{\text{acp53}}[\text{p53}] - k_{\text{dep53}}[\text{p53}^*] - k_{\text{dp53p}}[\text{Mdm2}_\text{n}]\frac{[\text{p53}^*]}{[\text{p53}^*] + j_{\text{1p53n}}} \quad (1)$$

$$\frac{d[\text{p53}]}{dt} = k_{\text{sp53}} - k_{\text{dp53n}}[\text{p53}] + k_{\text{dep53}}[\text{p53}^*] - k_{\text{acp53}}[\text{p53}] - k_{\text{dp53}}[\text{Mdm2}_\text{n}]\frac{[\text{p53}]}{[\text{p53}] + j_{\text{1p53n}}} \quad (2)$$

$$k_{\text{acp53}} = k_{\text{acp530}} + k_{\text{acp531}}\frac{\text{DD}}{\text{DD} + j_{\text{DDp53}}} \quad (3)$$

$$k_{\text{dmdm2n}} = k_{\text{dmdm2n0}} + k_{\text{dmdm2n1}}\frac{\text{DD}}{\text{DD} + j_{\text{DDm2}}} \quad (4)$$

$$\frac{d[\text{mdm2m}]}{dt} = k_{\text{smdm2m0}} + k_{\text{smdm2m}}\frac{[\text{p53}^*]^4}{[\text{p53}^*]^4 + j_{\text{smdm2m}}^4} - k_{\text{dmdm2m}}[\text{mdm2m}] \quad (5)$$

$$\frac{d[\text{Mdm2}_\text{n}]}{dt} = k_\text{i}[\text{Mdm2}_{\text{cp}}] - k_\text{o}[\text{Mdm2}_\text{n}] - k_{\text{dmdm2n}}[\text{Mdm2}_\text{n}] \quad (6)$$

$$\frac{d[\text{Mdm2}_\text{c}]}{dt} = k_{\text{tmdm2}}[\text{mdm2m}] - k_{\text{dmdm2c}}[\text{Mdm2}_\text{c}] + k_{\text{dpmdm2}}\frac{[\text{Mdm2}_{\text{cp}}]}{[\text{Mdm2}_{\text{cp}}] + j_{\text{dpmdm2}}} - k_{\text{pmdm2}}[\text{Akt}_\text{p}]\frac{[\text{Mdm2}_\text{c}]}{[\text{Mdm2}_\text{c}] + j_{\text{pmdm2}}} \quad (7)$$

$$k_{\text{tmdm2}} = \frac{k_{\text{tmdm20}}}{[\text{miR-192}] + j_{\text{DDm2}}} \quad (8)$$

$$\frac{d[\text{Mdm2}_{\text{cp}}]}{dt} = k_{\text{pmdm2}}[\text{Akt}_\text{p}]\frac{[\text{Mdm2}_\text{c}]}{[\text{Mdm2}_\text{c}] + j_{\text{pmdm2}}} - k_{\text{dpmdm2}}\frac{[\text{Mdm2}_{\text{cp}}]}{[\text{Mdm2}_{\text{cp}}] + j_{\text{dpmdm2}}} - k_\text{i}[\text{Mdm2}_{\text{cp}}] + k_\text{o}[\text{Mdm2}_\text{n}] - k_{\text{dmdm2c}}[\text{Mdm2}_{\text{cp}}] \quad (9)$$

$$\frac{d[\text{miR-192}]}{dt} = k_{\text{smiR1920}} + k_{\text{smiR192}} \frac{[\text{p53}^*]^4}{[\text{p53}^*]^4 + j_{\text{smiR192}}^4} - k_{\text{dmiR192}}[\text{miR-192}] \tag{10}$$

$$\frac{d[\text{Akt}_\text{p}]}{dt} = k_{\text{acakt}}[\text{PIP3}] \frac{[\text{Akt}]}{[\text{Akt}] + j_{\text{acakt}}} - k_{\text{deakt}} \frac{[\text{Akt}_\text{p}]}{[\text{Akt}_\text{p}] + j_{\text{deakt}}} \tag{11}$$

$$[\text{Akt}] = \text{Akt}_{\text{tot}} - [\text{Akt}_\text{p}] \tag{12}$$

$$\frac{d[\text{PIP3}]}{dt} = k_{\text{p2}} \frac{[\text{PIP2}]}{[\text{PIP2}] + j_{\text{p2}}} - k_{\text{p3}}[\text{PTEN}] \frac{[\text{PIP3}]}{[\text{PIP3}] + j_{\text{p3}}} \tag{13}$$

$$[\text{PIP2}] = \text{PIP}_{\text{tot}} - [\text{PIP3}] \tag{14}$$

$$\frac{d[\text{PTEN}]}{dt} = k_{\text{sPTEN0}} + k_{\text{sPTEN}} \frac{[\text{p53}^*]^4}{[\text{p53}^*]^4 + j_{\text{sPTEN}}^4} - k_{\text{dPTEN}}[\text{PTEN}] \tag{15}$$

$$\frac{d[\text{p21m}]}{dt} = k_{\text{sp21m0}} + k_{\text{sp21m}} \frac{[\text{p53}^*]^4}{[\text{p53}^*]^4 + j_{\text{sp21m}}^4} - k_{\text{dp21m}}[\text{p21m}] \tag{16}$$

$$\frac{d[\text{p21}_{\text{tot}}]}{dt} = k_{\text{tp21}}[\text{p21m}] - k_{\text{dp21}}[\text{p21}_{\text{tot}}] \tag{17}$$

$$k_{\text{tp21}} = \frac{k_{\text{tp210}}}{[\text{miR-22}] + j_{\text{22p21f}}} \tag{18}$$

$$k_{\text{dp21m}} = k_{\text{dp21m0}} + k_{\text{dp21m1}} \frac{[\text{miR-22}]}{[\text{miR-22}] + j_{\text{22p21d}}} \tag{19}$$

$$\frac{d[\text{miR-22}]}{dt} = k_{\text{smiR220}} + k_{\text{smiR22}} \frac{[\text{p53}^*]^4}{[\text{p53}^*]^4 + j_{\text{smiR22}}^4} - k_{\text{dmiR22}}[\text{miR-22}] \tag{20}$$

$$\begin{aligned}\frac{d[\text{p21CE}]}{dt} &= k_{\text{asp21ce}}[\text{p21}][\text{CycE}] - k_{\text{dsp21ce}}[\text{p21CE}] \\ &\quad -(k_{\text{dp21}} + k_{\text{dcyce}})[\text{p21CE}]\end{aligned} \tag{21}$$

$$[\text{p21}] = [\text{p21}_{\text{tot}}] - [\text{p21CE}] \tag{22}$$

$$\frac{d[\text{CycE}_{\text{tot}}]}{dt} = k_{\text{scyce0}} + k_{\text{scyce}} \frac{[\text{E2F1}]^2}{[\text{E2F1}]^2 + j_{\text{scyce}}^2} - k_{\text{dcyce}}[\text{CycE}_{\text{tot}}] \tag{23}$$

$$[\text{CycE}] = [\text{CycE}_{\text{tot}}] - [\text{p21CE}] \tag{24}$$

$$\begin{aligned}\frac{d[\text{E2F1}]}{dt} &= k_{\text{se2f1}} - k_{\text{asre}}[\text{Rb}][\text{E2F1}] + k_{\text{dsre}}[\text{RE}] \\ &\quad + k_{\text{prb}}[\text{CycE}] \frac{[\text{RE}]}{[\text{RE}] + j_{\text{prb}}} - k_{\text{de2f1}}[\text{E2F1}]\end{aligned} \tag{25}$$

$$\frac{d[\text{E2F1}_{\text{tot}}]}{dt} = k_{\text{se2f1}} - k_{\text{de2f1}}[\text{E2F1}_{\text{tot}}] \tag{26}$$

$$k_{\text{de2f1}} = k_{\text{de2f10}} + k_{\text{de2f11}} \frac{j_{\text{DD2f}}}{j_{\text{DD2f}} + \text{DD}} \tag{27}$$

$$[\text{RE}] = [\text{E2F1}_{\text{tot}}] - [\text{E2F1}] \tag{28}$$

$$\frac{d[\mathrm{Rb_p}]}{dt} = k_{\mathrm{prb}}[\mathrm{CycE}]\frac{[\mathrm{Rb}]}{[\mathrm{Rb}] + j_{\mathrm{prb}}} - k_{\mathrm{dprb}}\frac{[\mathrm{Rb_p}]}{[\mathrm{Rb_p}] + j_{\mathrm{dprb}}} + k_{\mathrm{prb}}[\mathrm{CycE}]\frac{[\mathrm{RE}]}{[\mathrm{RE}] + j_{\mathrm{prb}}} \quad (29)$$

$$[\mathrm{Rb}] = [\mathrm{Rb_{tot}}] - [\mathrm{Rb_p}] - [\mathrm{RE}] \quad (30)$$

$$\frac{d[\mathrm{Bax}]}{dt} = k_{\mathrm{sbax0}} + k_{\mathrm{sbax}}\frac{[\mathrm{p53^*}]^4}{[\mathrm{p53^*}]^4 + j_{\mathrm{sbax}}^4} - k_{\mathrm{dbax}}[\mathrm{Bax}] \quad (31)$$

$$\frac{d[\mathrm{CytoC}]}{dt} = (k_{\mathrm{accytoc0}} + k_{\mathrm{accytoc1}}[\mathrm{Bax}]\frac{[\mathrm{Casp3}]^4}{[\mathrm{Casp3}]^4 + j_{\mathrm{cap3bx}}^4})(\mathrm{CytoC_{tot}} - [\mathrm{CytoC}]) - k_{\mathrm{decytoc}}[\mathrm{CytoC}] \quad (32)$$

$$\frac{d[\mathrm{Apaf1}]}{dt} = k_{\mathrm{sapaf10}} + k_{\mathrm{sapaf1}}\frac{[\mathrm{E2F1}]^2}{[\mathrm{E2F1}]^2 + j_{\mathrm{sapaf1}}^2} - k_{\mathrm{dapaf1}}[\mathrm{Apaf1}] \quad (33)$$

$$\frac{d[\mathrm{Apops}]}{dt} = k_{\mathrm{acapops}}(([\mathrm{CytoC}] - 7[\mathrm{Apops}])([\mathrm{Apaf1}] - 7[\mathrm{Apops}]))^7 - k_{\mathrm{deapops}}[\mathrm{Apops}] \quad (34)$$

$$\frac{d[\mathrm{Casp9}]}{dt} = (k_{\mathrm{accasp90}} + k_{\mathrm{accasp91}}\frac{[\mathrm{Casp3}]^4}{[\mathrm{Casp3}]^4 + j_{\mathrm{casp3}}^4}\frac{[\mathrm{Apops}]^4}{[\mathrm{Apops}]^4 + j_{\mathrm{apops}}^4})(\mathrm{Casp9_{tot}} - [\mathrm{Casp9}]) - k_{\mathrm{decasp9}}[\mathrm{Casp9}] \quad (35)$$

$$\frac{d[\mathrm{Casp3}]}{dt} = (k_{\mathrm{accasp30}} + k_{\mathrm{accasp31}}\frac{[\mathrm{Casp9}]^4}{[\mathrm{Casp9}]^4 + j_{\mathrm{casp9}}^4})(\mathrm{Casp3_{tot}} - [\mathrm{Casp3}]) - k_{\mathrm{decasp3}}[\mathrm{Casp3}] \quad (36)$$

## Supplemental Table S1: Variables and their initial values

| Variable | initial values | Variable | initial values | Variable | initial values | Variable | initial values |
|---|---|---|---|---|---|---|---|
| [p53*] | 0.0009 | [miR-192] | 0.1 | [miR-22] | 0.1 | [Bax] | 0.1 |
| [p53] | 0.0343 | [$\mathrm{Akt_p}$] | 0.9415 | [p21CE] | 0.08 | [CytoC] | 0.197 |
| [mdm2m] | 0.05 | [PIP3] | 0.8944 | [$\mathrm{CycE_{tot}}$] | 0.166 | [Apaf1] | 0.01 |
| [$\mathrm{Mdm2_n}$] | 0.6413 | [PTEN] | 0.1 | [E2F1] | 0.1 | [Apops] | 0 |
| [$\mathrm{Mdm2_c}$] | 0.0314 | [p21m] | 0.15 | [$\mathrm{E2F1_{tot}}$] | 1 | [Casp9] | 0.0588 |
| [$\mathrm{Mdm2_{cp}}$] | 0.9939 | [$\mathrm{p21_{tot}}$] | 0.188 | [$\mathrm{Rb_p}$] | 0 | [Casp3] | 0.0727 |

# Supplemental Table S2: Parameter Values

| Parameter | Description | Value | Reference |
|---|---|---|---|
| $j_{DDp53}$ | Michaelis constant of DNA damage-dependent p53 activation | 20 | estimated |
| $j_{DDm2}$ | Michaelis constant of DNA damage-dependent nuclear Mdm2 degration | 22 | estimated |
| $k_{smdm20}$ | Basal induction rate of mdm2 mRNA | 0.002 | [1] |
| $k_{smdm2}$ | p53-dependent transcription rate of mdm2 | 0.02 | [1,3] |
| $j_{smdm2m}$ | Michaelis constant of p53-dependent mdm2 mRNA production | 1 | [2,3] |
| $k_{dmdm2m}$ | Degradation rate of mdm2 mRNA | 0.04 | estimated |
| $k_{tmdm20}$ | MiR-192-dependent translation rate of mdm2 mRNA | 0.2 | estimated |
| $j_{mir192}$ | Michaelis constant of miR-192-induced mdm2 mRNA translation repression | 0.1 | estimated |
| $k_{dmdm2n0}$ | Basel degradation rate of nuclear Mdm2 | 0.03 | [3,4] |
| $k_{dmdm2n1}$ | DNA damage-dependent degradation rate of nuclear Mdm2 | 0.5 | estimated |
| $k_{dmdm2c}$ | Degradation rate of cytoplasmic Mdm2 | 0.03 | [4] |
| $k_{dpmdm2}$ | Dephosphorylation rate of cytoplasmic Mdm2 | 0.3 | [3] |
| $j_{dpmdm2}$ | Michaelis constant of Mdm2 dephosphorylation | 0.1 | [2,3] |
| $k_{pmdm2}$ | Akt-dependent phosphorylation rate of cytoplasmic Mdm2 | 3.6 | [3] |
| $j_{pmdm2}$ | Michaelis constant of Akt-dependent Mdm2 phosphorylation | 0.3 | [2,3] |
| $k_i$ | Nuclear import rate of $Mdm2_{cp}$ | 0.6 | [2] |
| $k_o$ | Nuclear export rate of $Mdm2_n$ | 0.9 | [2] |
| $k_{smiR1920}$ | Basal induction rate of miR-192 | 0.0008 | estimated |
| $k_{smiR192}$ | p53-dependent induction rate of miR-192 | 0.01 | estimated |
| $j_{smiR192}$ | Michaelis constant of p53-dependent miR-192 production | 0.4 | estimated |
| $k_{dmiR192}$ | Degradation rate of miR-192 | 0.008 | Estimated |
| $k_{acp531}$ | DNA damage-dependent activation rate of p53 | 0.016 | estimated |
| $k_{acp530}$ | Basal activation rate of p53 | 0.0016 | estimated |
| $k_{dep53}$ | Deactivation rate of p53 | 0.008 | estimated |
| $k_{dp53p}$ | Mdm2-dependent degradation rate of $p53_p$ | 0.008 | [1] |
| $k_{sp53}$ | Production rate of p53 | 0.08 | [1,3] |
| $k_{dp53n}$ | Basal degradation rate of p53 | 0.04 | [1,3] |
| $k_{dp53}$ | Mdm2-dependent degradation rate of p53 | 0.48 | [1] |
| $j_{1p53n}$ | Michaelis constant of Mdm2-dependent p53 degradation | 0.1 | [2,3] |
| $k_{acakt}$ | Phosphorylation rate of Akt | 0.25 | [2,3] |
| $j_{acakt}$ | Michaelis constant of Akt phosphorylation | 0.1 | [2,3] |
| $k_{deakt}$ | Dephosphorylation rate of $Akt_p$ | 0.1 | [2,3] |
| $j_{deakt}$ | Michaelis constant of $Akt_p$ dephosphorylation | 0.2 | [2,3] |
| $k_{p2}$ | Phosphorylation rate of PIP2 | 0.1 | [2] |

| | | | |
|---|---|---|---|
| $j_{p2}$ | Michaelis constant of PIP2 phosphorylation | 0.2 | [2] |
| $k_{p3}$ | PTEN-dependent dephosphorylation rate of PIP3 | 0.5 | [2] |
| $j_{p3}$ | Michaelis constant of PIP3 dephosphorylation | 0.4 | [2] |
| $PIP_{tot}$ | Total concentration of PIP2 and PIP3 | 1 | [2,3] |
| $Akt_{tot}$ | Total concentration of Akt | 1 | [2,3] |
| $k_{sPTEN0}$ | Basal induction rate of PTEN | 0.001 | [5] |
| $k_{sPTEN}$ | p53-inducible production rate of PTEN | 0.06 | [5] |
| $j_{sPTEN}$ | Michaelis constant of p53-dependent PTEN production | 2.5 | [5] |
| $k_{dPTEN}$ | Degradation rate of PTEN | 0.01 | [5] |
| $k_{sp21m0}$ | Basal induction rate of p21 mRNA | 6.8*E-5 | estimated |
| $k_{sp21m}$ | p53-dependent transcription rate of p21 | 0.0035 | estimated |
| $j_{sp21m}$ | Michaelis constant of p53-dependent p21 mRNA production | 0.75 | [5] |
| $k_{dp21m0}$ | Basal degradation rate of p21 mRNA | 7.5*E-5 | estimated |
| $k_{dp21m1}$ | MiR-22-dependent degradation rate of p21 mRNA | 0.003 | estimated |
| $j_{22p21d}$ | Michaelis constant of miR-22-dependent p21 mRNA degradation | 0.7 | estimated |
| $k_{tp210}$ | MiR-22-dependent translation rate of p21 mRNA | 0.01 | estimated |
| $j_{22p21f}$ | Michaelis constant of miR-22-induced p21 mRNA translation repression | 0.3 | estimated |
| $k_{dp21}$ | Degradation rate of p21 | 0.02 | [6] |
| $k_{smiR220}$ | Basal induction rate of miR-22 | 0.0006 | estimated |
| $k_{smiR22}$ | p53-dependent induction rate of miR-22 | 0.0075 | estimated |
| $j_{smiR22}$ | Michaelis constant of p53-dependent miR-22 production | 1.9 | estimated |
| $k_{dmiR22}$ | Degradation rate of miR-22 | 0.006 | estimated |
| $k_{asp21ce}$ | Combining rate of p21 and CycE | 2 | estimated |
| $k_{dsp21ce}$ | Dissociation rate of p21-CycE complex | 0.1 | estimated |
| $k_{scyce0}$ | Basal induction rate of CycE | 0.01 | estimated |
| $k_{scyce}$ | E2F1-dependent induction rate of CycE | 0.15 | estimated |
| $j_{scyce}$ | Michaelis constant of E2F1-dependent CycE production | 0.4 | estimated |
| $k_{dcyce}$ | Degradation rate of CycE | 0.06 | estimated |
| $k_{asre}$ | Combining rate of Rb and E2F1 | 0.8 | estimated |
| $k_{dsre}$ | Dissociation rate of Rb-E2F1 complex | 0.1 | estimated |
| $k_{se2f1}$ | Production rate of E2F1 | 0.0005 | estimated |
| $k_{de2f10}$ | Basal degradation rate of E2F1 | 6*E-5 | [7] |
| $k_{de2f11}$ | DNA damage-dependant degradation rate of E2F1 | 4.4*E-4 | [7] |
| $j_{DD2f}$ | Michaelis constant of DNA damage-induced E2F1 degradation repression | 60 | estimated |
| $k_{prb}$ | Phosphorylation rate of Rb by CycE | 0.4 | estimated |
| $j_{prb}$ | Michaelis constant of Rb phosphorylation by CycE | 3.9 | estimated |
| $k_{dprb}$ | Dephosphorylation rate of Rb | 0.2 | estimated |

| | | | |
|---|---|---|---|
| $j_{dprb}$ | Michaelis constant of Rb dephosphorylation | 1 | estimated |
| $[Rb_{tot}]$ | Total concentration of Rb | 2 | estimated |
| $k_{sbax0}$ | Basal induction rate of Bax | 0.002 | estimated |
| $k_{sbax}$ | p53-dependent induction rate of Bax | 0.06 | estimated |
| $j_{sbax}$ | Michaelis constant of p53-dependent Bax production | 2.3 | estimated |
| $k_{dbax}$ | Degradation rate of Bax | 0.02 | estimated |
| $k_{accytoc0}$ | Basal release rate of mitochondrial cytochrome c | 0.001 | [2] |
| $k_{accytoc1}$ | Bax-dependent release rate of mitochondrial cytochrome c | 1.5 | [2] |
| $j_{cap3bx}$ | Michaelis constant of caspase-3 dependent cytochrome c release | 0.2 | [2] |
| $k_{decytoc}$ | Mitochondrial influx rate of cytochrome c | 0.05 | [2] |
| $Cytoc_{tot}$ | Total concentration of cytochrome c | 3 | [2] |
| $k_{sapaf10}$ | Basal induction rate of Apaf-1 | 0.001 | [5] |
| $k_{sapaf1}$ | E2F1-dependent induction rate of Apaf-1 | 0.25 | [5] |
| $j_{sapaf1}$ | Michaelis constant of E2F1-dependent Apaf-1 production | 0.8 | [5] |
| $k_{dapaf1}$ | Degradation rate of Apaf-1 | 0.1 | [5] |
| $k_{acapops}$ | Rate of Apaf-1 and cytochrome c assembling into apoptosome | 0.7 | estimated |
| $k_{deapops}$ | Deactivation rate of apoptosome | 0.5 | [5] |
| $Casp9_{tot}$ | Total concentration of caspase-9 | 3 | [5] |
| $k_{accasp90}$ | Basal activation rate of caspase-9 | 0.001 | [5] |
| $k_{accasp91}$ | Caspase-3- and Apoptosome-dependent activation rate of caspase-9 | 3 | [5] |
| $j_{casp3}$ | Michaelis constant of caspase-3-dependent caspase-9 activation | 0.3 | estimated |
| $j_{apops}$ | Michaelis constant of apoptosome-dependent caspase-9 activation | 0.35 | estimated |
| $k_{decasp9}$ | Deactivation rate of caspase-9 | 0.05 | [5] |
| $Casp3_{tot}$ | Total concentration of caspase-3 | 3 | [5] |
| $k_{accasp30}$ | Basal activation rate of caspase-3 | 0.001 | [5] |
| $k_{accasp31}$ | Caspase-9-dependent activation rate of caspase-3 | 0.5 | estimated |
| $j_{casp9}$ | Michaelis constant of caspase-9-dependent caspase-3 activation | 0.3 | estimated |
| $k_{decasp3}$ | Deactivation rate of caspase-3 | 0.07 | [5] |


[1] Ma, L., J. Wagner, J. J. Rice, W. Hu, A. J. Levine, and G. A. Stolovitzky. 2005. A plausible model for the digital response of p53 to DNA damage. *Proc. Natl. Acad. Sci. USA.* 102:14266-14271.

[2] Zhang, X. P., F. Liu, and W. Wang. 2011. Two-phase dynamics of p53 in the DNA damage response. *Proc. Natl. Acad. Sci. USA.* 108:8990-8995.

[3] Wee, K. B., and Aguda, B. D. 2006. Akt versus p53 in a network of oncogenes and tumor suppressor genes regulating cell survival and death. Biophys. J. 91, 857-865.

[4] Stommel, J. M., and G. M. Wahl. 2004. Accelerated MDM2 autodegradation induced by DNA-damage kinases is required for p53 activation. *EMBO J.* 23:1547-1556

[5] Li H, Zhang X-P, Liu F. 2013. Coordination between p21 and DDB2 in the Cellular Response to UV Radiation. *PLoS ONE* 8(11): e80111

[6] Maki CG and Howley PM. (1997). Ubiquitination of p53 and p21 Is Differentially Affected by Ionizing and UV Radiation Mol. Cell. Biol., 17, 355-363.

[7] Qi Xie, Yujie Bai, et al. (2011). Methylation-mediated regulation of E2F1 in DNA damage induced cell death. Journal of Receptors and Signal Transduction., 31(2): 139–146

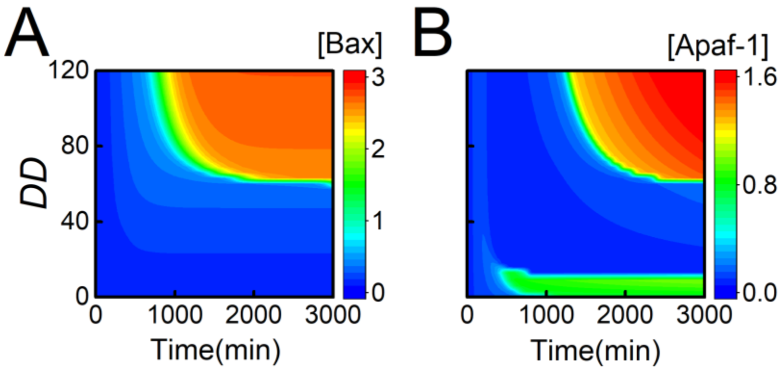


Figure S1: Heat map of [Bax] (*A*) and [Apaf-1] (*B*) as a function of Time and *DD*.
